# Nanoscale Silicon Fingerprints for Counterfeit Prevention in Microchips

**DOI:** 10.1002/smll.202500878

**Published:** 2025-02-04

**Authors:** Bo Liu, Amin Farhadi, Theresa Bartschmid, Yamin Zhang, Chunsheng Guo, Shiwei Feng, Gilles R. Bourret

**Affiliations:** ^1^ Faculty of Information Technology College of Microelectronics Beijing University of Technology Beijing 100124 P. R. China; ^2^ Department of Chemistry and Physics of Materials University of Salzburg Salzburg A‐5020 Austria

**Keywords:** chip anti‐counterfeiting, dewetting, metal‐assisted chemical etching, physical unclonable function, shapley value

## Abstract

The increasing vulnerability of microchips to counterfeiting poses a significant threat to nations, companies, and the general public. Creating a unique “fingerprint” on each chip using intrinsic manufacturing variations can significantly prevent the number of fraudulent chips. Since Si‐based semiconductor fabrication processes are now flawless down to a few nanometers, finding a high‐entropy source at the nanoscale has become challenging. Inspired by the concept of physical unclonable function, this work reports the CMOS‐compatible and lithography‐free fabrication of unique nanostructured silicon “fingerprints.” Nanostructuring is achieved via low‐temperature dewetting and metal‐assisted chemical etching, which produces a high level of entropy and unique silicon‐based nanoscale fingerprints with linewidths tunable from ≈8 to 140 nm, commensurate with the dimensions of mainstream microfabrication processes. These Si nanofingerprints are highly reliable for chip authentication and against reverse engineering, providing a large encoding capacity of up to 2^16384^/µm^2^. For practical applications, detection of fingerprints protected with a polymer coating is demonstrated using back‐scattered electron imaging.

## Graphical abstract

The low‐cost, CMOS‐compatible, and lithography‐free synthesis of unique silicon fingerprints with encoding capacity up to 2^16384^/µm^2^ is reported using metal‐assisted chemical etching and low temperature thermal dewetting of metal films. The method provides an optimum 50% coverage of nanostructured silicon, a high level of randomness, and linewidths down to 8 nm, making it resilient against machine learning attacks.

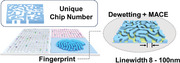

## 1 Introduction

The threat of counterfeit chips is often underestimated. When integrated into essential electronic systems, such as medical devices, financial transaction platforms, self‐driving vehicles, or military and aerospace technologies, counterfeit chips can pose a substantial risk to the security of information, the global economy, and even public health. Because fraudulent chips are sourced from electronic waste and only undergo crude re‐marking processes before being reintroduced into the market, they have much higher failure rates than genuine ones. To address the dangers posed by counterfeit chips, the World Semiconductor Council (WSC) established an anti‐counterfeiting task force (ACTF) department in 2012 (more details in Note SI, Supporting Information). Under this framework, the Semiconductor Industry Association (SIA) released a white paper, titled “Winning the Battle Against Counterfeit Semiconductor Products” to bring awareness to the increasing challenges that these counterfeit chips present.^[^ ^1^ ^]^ One decade has passed and relatively little progress has been made to‐date, as reported by the Electronics Research and Analysis Institute (ERAI).^[^ ^2^ ^]^ As a result, the number of counterfeit chips released into the market is still increasing.

Unlike most products, chips can be mishandled without clear evidence of abuse. Efficient anti‐counterfeit techniques such as a secure split test (SST), hardware metering, and hardware intrinsic security (HIS) have been developed to track each chip from their original component manufacturers (OCMs) to authorised selling sources and to the terminal systems (more information on the definition, taxonomy, detection and avoidance techniques of the counterfeited chip can be found in the Figure S1 and Notes SII–SV, Supporting Information).^[^ ^3^, ^4^ ^]^ SST protect chips from cloning and overproduction, while hardware metering provides each chip with a unique tag, which can be used for tracing.^[^ ^5^ ^]^ To provide unique and unpredictable tags for tracking each chip, physical unclonable functions (PUFs) have emerged as an efficient alternative.^[^ ^6^ ^]^ PUF belongs to the HIS category and can be used to generate a unique “fingerprint” for each electronic component based on their inherent manufacturing variations.^[^ ^7^ ^]^ Such fingerprints are highly unpredictable and resistant to tampering.

Since microchips are predominantly composed of silicon, creating unique identifiers from silicon appears as a practical solution. Si‐based fingerprints should meet the following technical specifications and be: i) Compatible with current microfabrication processes: The synthesis of the Si‐based PUF should be CMOS‐compatible, and as easy as possible; ii) Space‐friendly: The PUF linewidth should be within current CMOS‐node range (e.g., 7/14 nm node) to reduce the space taken by the chip overhead and the residue function regions; iii) Detectable and with large encoding capacity: The challenge‐response pairs (CRPs) should be detectable and the CRP space should be large enough for commercial use; iv) Offers a high degree of randomness: The entropy source should be highly stochastic, with an equal probability of “1” or “0,” unclonable and unpredictable, e.g. the PUF should be resilient to reverse engineering, model attacks and machine learning attacks.

Electronic‐ and photonic‐based strategies could potentially be used to authenticate Si microchips. Electronic‐based PUFs could be well‐matched with a wide range of CMOS nodes. However, semiconductor microfabrication processes are now flawless down to a few nanometers: Finding a high‐entropy source at the nanoscale has become challenging. Emerging CMOS‐compatible nanotechnologies could provide plenty of entropy sources,^[^ ^8^, ^9^ ^]^ but they have suffered from high production costs and some unsolved technical issues which has prevented their use in industry (see Note SVI, Supporting Information for a brief introduction to electronic PUFs^[^ ^7^, ^10^ ^]^). Alternatively, unique structured surfaces, made of proteins,^[^ ^11^ ^]^ polymers,^[^ ^12^, ^13^ ^]^ metals,^[^ ^14^ ^]^ or liquid crystals,^[^ ^15^ ^]^ have been developed to generate unique optical patterns. Such optical/photonic PUFs have recently been proposed as a solution for authenticating arbitrary substrates.^[^ ^13^, ^14^ ^]^ However, these approaches are either limited to the production of large features that are not space efficient,^[^ ^14^ ^]^ have not been tested at temperatures > 150 °C,^[^ ^13^ ^]^ or need to be prepared after the microchip fabrication and packaging process,^[^ ^13^, ^15^ ^]^ which is not fully compatible with CMOS technology. To‐date, the development of robust, CMOS‐compatible, cost‐effective, space‐efficient and unpredictable Si‐based PUF has become essential to address the global threat posed by counterfeit chips. This remains a challenging task.

**Figure 1:**
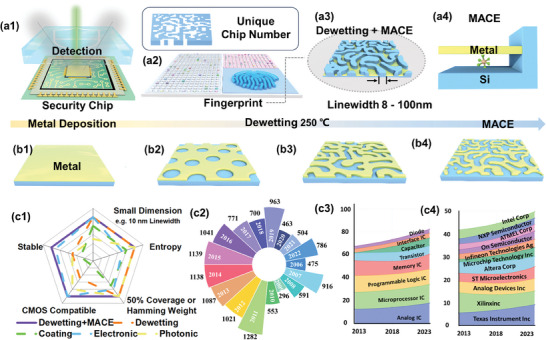
Si nanofingerprint for chip authentification. Schematic depiction of: a1,a2) Si nanofingerprint integrated within a chiplet and detected via backscattered electron microscopy; a3) Enlarged view of the Si nanofigerprint after MACE before the final metal film dissolution step, where the blue region is Si and yellow region is the metal (Au or Au/Ag); a4) Illustration of metal‐assisted chemical etching (MACE); b1–b4) Schematic showing the evolution of the metal film morphology during dewetting. c1) Radar map comparing the Si nanofingerprints produced via dewetting/MACE with existing methods for chip anti‐counterfeiting. c2‐c4) Counterfeit chip reported data, taxonomy, detection methods and avoidance techniques. c2) Reported counterfeited chip each year, ranging from 2006 to 2022; c3) Proportion of the device type of the reported counterfeited chip from 2013 to 2023 (cases below 3.5% proportion are not included); c4) the proportion of the marking logo of the reported counterfeited chip from 2013 to 2023 (cases below 2% proportion are not included); The dataset shown in (c2–c4) is collected from the public report of ERAI.

We report here the fabrication and study of nanoscale Si‐based randomly organized structures for chip authentication, which can be detected via back‐scattered electron (BSE) imaging underneath a protecting polymer coating (**Figure** **1**‐a1,a2). Si nanostructuring is achieved via sequential dewetting‐assisted patterning, which generates metal holey meshes with tunable morphologies, used as catalytic etching masks for metal‐assisted chemical etching (MACE)^[^ ^16^ ^]^ (Figure 1‐a3,a4). MACE is a low‐cost, bench‐top, simple, and fast technique that affords the precise, CMOS‐compatible and highly anisotropic etching of silicon.^[^ ^17^, ^18^ ^]^ Compared to conventional microfabrication methods such as reactive ion etching, MACE does not require expensive equipment, provides smoother sidewalls, and is compatible with the preparation of ultra‐high aspect ratio nanostructures.^[^ ^18^ ^]^ Thus, MACE has been used for a variety of applications ranging from nanoelectronics, nanophotonics, chemical and bio‐sensing, biomedicine to solar conversion.^[^ ^18^, ^19^, ^20^ ^]^ Despite its high versatility and remarkable spatial resolution, MACE has not been used to develop PUFs for chip anti‐counterfeiting. The inherent randomness of the thermal dewetting step used to nanostructure the MACE mask leads to a high entropy and unique silicon‐based nanoscale fingerprint, with a linewidth tunable from ≈8 to 140 nm. Such nanoscale features are easily accessible using thermal dewetting and MACE.^[^ ^16^ ^]^ High entropy Si‐based PUFs with a large CRP space of up to 2^16384^ are produced within regions as small as 1 µm^2^, which can be used for single‐chip authentication. The synthesis includes only four steps, is CMOS‐compatible, and resilient against machine learning attacks. For practical applications, the PUF linewidth under the current CMOS node ranges should still be detectable after packaging. Advanced ptychographic X‐ray computed tomography (PXCT) can provide a 3D tomogram with a resolution down to 14.6 nm, e.g., visualisation of the inner circuit design of the Intel Pentium G3260 processor with 22 nm technology.^[^ ^21^, ^22^ ^]^ As such, PXCT is well‐suited for identifying Si fingerprints under the packaging resin. However PXCT requires advanced instrumentation and data treatment, which is not yet widely available.^[^ ^21^, ^22^ ^]^ We report here a more convenient and cost‐friendly alternative detection method based on BSE imaging within a scanning electron microscope (SEM). Identification of Si fingerprints buried under a protecting polymer coating is demonstrated via standard BSE imaging (details in Section BSE Detection), which highlights the feasibility to physically protect the Si fingerprint without compromising its detection using standard methods.

The synthesis of the Si nanofingerprints via sequential dewetting and MACE is depicted in Figure 1‐a3,a4, and b1–b4. Solid state dewetting yields randomly nanostructured metal thin films,^[^ ^23^ ^]^ used as masks for the subsequent MACE step.^[^ ^16^ ^]^ MACE is based on the catalytic reduction of a strong oxidant (here, hydrogen peroxide, H_2_O_2_) at the surface of the metal film that injects holes through the metal/silicon Schottky junction, which, in the presence of HF leads to the dissolution of Si as SiF_6_^2−^, a water‐soluble product.^[^ ^18^ ^]^ Because the reduction of H_2_O_2_ is kinetically hindered at the silicon surface, the silicon preferentially dissolves only in the regions that are covered by the metal film. During MACE, nanostructured metal films keep intimate contact with the silicon surface thanks to strong van der Waals interactions, thus providing a strongly anisotropic etching: The metal film pattern is transferred to the silicon substrate with very high fidelity. Since MACE is highly selective and can be used to structure silicon down to the nanometer scale, the randomness of the metal pattern used to synthesize the Si nanofingerprint is key (see molecular dynamic simulations presented in the next section). To prevent the formation of surface defects during MACE on silicon wafers caused by HF exposure, the process could be confined to the Si fingerprint region by protecting the rest of the wafer with a temperature‐stable photoresist. Additionally, HF etching, when done under controlled conditions, is a standard technique in the microfabrication industry that is CMOS‐compatible. Therefore, our approach has the potential to integrate seamlessly into the microchip manufacturing process.

Figure 1‐c1 compares existing PUF technologies with our combined dewetting/MACE strategy in the context of microchip anticounterfeiting. The ability of each PUFs‐system to provide small feature dimensions (i.e., linewidths), an optimum Hamming weight (i.e., equal to 0.5), a high level of entropy value, a high CMOS‐compatibility and thermal/environmental stability is summarized. In short, the most popular PUF‐systems suffer from the following constraints: Surface coatings^[^ ^12^, ^24^ ^]^ are neither stable nor secure because they need to be applied after packaging; Photonic approaches rely on rough surfaces,^[^ ^15^ ^]^ which needs to be produced at the surface of the chip after packaging, and are thus not particularly stable; Electronic‐based methods are highly compatible with the entire CMOS process chain but provide limited entropy due to the near‐flawless fabrication process,^[^ ^7^ ^]^ while nanotechnology‐based electronic devices are still not space efficient,^[^ ^10^ ^]^ and as such are not taken into account on this map; Low temperature dewetting of metal thin films^[^ ^16^ ^]^ can generate ultra‐thin linewidths, which, without a transfer into silicon via MACE, will dewet further during the later microchip fabrication steps: Without MACE, dewetting produces unstable PUFs. Instead, high‐temperature dewetting (i.e., in the 500–900 °C range) of Au films on SiO_2_ has been investigated as an alternative solution to provide relatively high‐temperature stability.^[^ ^14^ ^]^ However, such high temperatures are not CMOS‐compatible. Additionally, the technique provided produces large Au‐based PUFs with average linewidths >500 nm, minimum encoding surface areas in the >1000 µm^2^ range, and sub‐optimal coverage rates that are much lower than 50%.^[^ ^14^ ^]^ Additionally, As shown in Figure 1c, our combined dewetting/MACE strategy currently provides the most promising PUF‐based anticounterfeiting technology platform for silicon microchips, surpassing current strategies on all five metrics presented and addressing the several challenges of counterfeited chip prevention as shown in Figure 1‐c2–c4, which are all collected by the ERAI reports, including the numbers, device types and remarking logos of the counterfeited chips.^[^ ^2^ ^]^

## 2 Metal Dewetting

The metastability of thin metal films can cause solid‐state dewetting at temperatures well below the melting temperature, leading to dramatic changes in the film morphology.^[^ ^23^ ^]^ Thus, metal thin film instability has long plagued the OCM of ICs that require well‐defined metal patterns. As a result, a variety of approaches have been developed to address this critical issue, such as the use of higher melting temperature metals and thicker films, the release of the residual stress in the as‐deposited films, improvement in film deposition quality to avoid the formation of defects and dislocations, and engineering of the metal‐substrate interface energy. Instead, we explore here the metal thin film instability as a valuable entropy source to synthesize unique Si‐based nano PUFs for chip authentication. The metal films are removed after Si nanostructuring to avoid further dewetting, reflowing, or any other detrimental effect that could be caused by the metal film to the IC performances during the following high‐temperature processing steps.

Minimization of the surface and interfacial energies can drive solid‐state dewetting at surprisingly low temperatures.^[^ ^23^ ^]^ As‐deposited metal thin films usually dewet with the following sequence: hole formation, hole growth and eventually film rupture.^[^ ^23^ ^]^ The resulting mass transport can produce a variety of nanoscale openings in the polycrystalline metal film, leading to metal patterns with tunable morphologies and dimensions.^[^ ^16^ ^]^ The formation and evolution of such patterns arise from local instabilities at the nanoscale, which are influenced by local variations in film thickness, microstructure, and thermal fluctuations that cannot be predicted or experimentally controlled. As such, dewetting produces random patterns.

**Figure 2:**
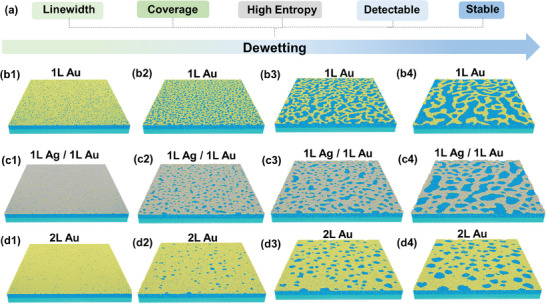
Molecular dynamics simulations of the metal film dewetting process. a) The essential parameters of Si nanofingerprints prepared via dewetting; b–d) MD simulations showing the time evolution of the metal film morphology during thermal dewetting at 250 °C on a SiO_2_/Si substrate for different film thicknesses and compositions: b1–b4) 1 monolayer of Au atoms, c1–c4) 1 monolayer of Ag atoms on top of 1 monolayer of Au atoms, and d1–d4) 2 monolayers of Au atoms. Time step: 1 fs.

The inherently random dewetting process is the key to forming unique serpentine patterns and is the entropy source of these Si‐based PUFs. We use here the rapid dewetting of Au and Au/Ag thin films at 250 °C to control the metal film morphology down to the sub‐10 nm scale, which is then transferred into silicon via MACE.^[^ ^16^ ^]^ As shown in **Figure** **2**a, in order to create high quality Si‐based fingerprint, the following critical parameters should be considered when optimizing dewetting conditions: i) The metal linewidth should be as small as possible to reduce the Si nanostructure footprint; ii) The metal coverage should be ideally 50% to obtain equal probability of generation “1” or “0”; iii) The pattern generation should be a high entropy source to prevent potential attacks and provide enough encoding capacity for commercial uses; iv) The metal pattern resulting after dewetting should be stable to undergo MACE without significant delamination, thus providing an efficient transfer of the pattern into the silicon substrate to enable detection at a later stage.

Molecular dynamic (MD) simulations were performed (Figure 2b–d) to investigate the morphological changes of Au and AuAg thin films perforated with random vacancy defects at 250 °C (substrate: SiO_2_, **Table** **1** summarizes the parameters used, more details in Methods). Our MD simulations show that the thinnest gold film dewets into serpentine patterns (Figure 2‐b1–b4), while the more stable thicker gold film only dewets to form a holey mesh (Figure 2‐d1–d4). The Ag/Au film after dewetting exhibits a similar serpentine pattern with a thicker linewidth (Figure 2‐c1–c4). These simulation results qualitatively match the morphology of the Au and Ag/Au films we produced after thermal dewetting, in agreement with previous thermal dewetting studies of similar metal thin films.^[^ ^16^, ^25^ ^]^ The time evolution of the local tension during dewetting is shown in Figures S3 and S4 (Supporting Information).

**Table 1:** Parameters used for the MD simulations.

	Ag─O	Ag─Si	Ag─Au	Au─O	Au─Si
ε (eV)	0.0592	0.0776	0.3903	0.0670	0.0877
σ (Å)	3.0246	3.3699	2.6405	3.0206	3.3654

## 3 The Si‐Based Fingerprint

**Figure 3:**
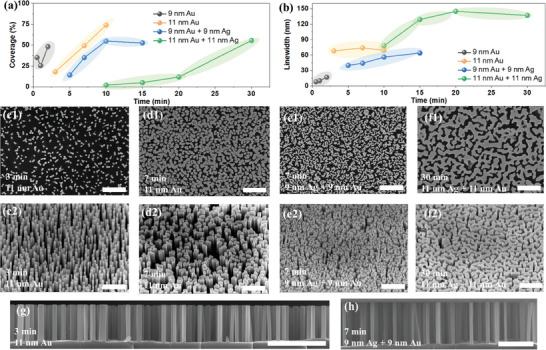
Si nanofingerprint morphology and coverage. a,b) Influence of dewetting duration on nanostructured Si coverage (a), and linewidth (b) obtained after thermal dewetting and MACE of: a 9 nm Au film (black line and symbol), an 11 nm Au film (orange line and symbol), a 9 nm Au/Ag film (blue line and symbol), and a 11 nm Au/Ag film (green line and symbol). Selected top‐view c1,d1,e1,f1) and tilted view c2,d2,e2,f2) SEM images, and cross‐section SEM images g,h) of the resulting Si nanofingerprints. c–h) Scale bars are 1µm.

Au and Au/Ag bilayer films dewet on silicon surfaces at 250 °C to form nanostructured metal films with a variety of morphologies, which depend on film composition and thickness, and annealing duration.^[^ ^16^ ^]^ Four different Au and Au/Ag films were deposited on clean Si substrates via sputtering, as described previously: 9 nm Au, 11 nm Au, 9 nm Au + 9 nm Ag, and 11 nm Au + 11 nm Ag (more details in the Methods section). Dewetting was performed at 250 °C in air for durations ranging from 30 s to 30 min, followed by MACE in an HF/H_2_O_2_ aqueous solution for several minutes.^[^ ^16^, ^26^ ^]^ Note that for practical application, the dewetting is better carried out in a vacuum environment to avoid potential contaminations on the Si surface. The nanostructured silicon coverage (i.e., the ratio between the nanostructured surface area divided by the corresponding sample surface area, expressed in %) and average Si nanostructure linewidth are extracted from each sample's scanning electron microscope (SEM) images, and shown in **Figure** **3**a,b. SEM images of some selected samples are shown in Figure 3c–h. More SEM images are provided in Figures S5 and S6 (Supporting Information). Overall, longer dewetting durations lead to increased linewidths and coverages, tunable from ≈8 to 140 nm, and 5% to 75%, respectively. For chip authentication, the encoding capacity of our Si‐based PUF is maximized when the nanostructured Si coverage is 50%, corresponding to a 50% response probability of “1” and “0,” and the Si nanofingerprint linewidth is the smallest, yielding the highest information density. In line with previous results,^[^ ^16^ ^]^ we find that: The 9 nm thick Au film provides the smallest linewidths (i.e., 8 nm), achieving a 17 nm linewidth with a ≈50% coverage rate after 2 min of dewetting; The 11 nm thick Au film yields a stable 70 nm lindewidth at all dewetting durations investigated (i.e., up to 10 min), but with a wide variability in nanostructured Si coverage, which ranges from ≈25 to 75%; The 9 nm Au/Ag bilayer film shows a stable 50% coverage for various dewetting durations between 10–15 min, e.g. it is the most robust at maintaining a 50% coverage under our experimental conditions; The 11 nm Au/Ag bilayer film requires much longer dewetting time to reach 50% coverage and forms the largest linewidth of ≈140 nm. Thus, it is possible to adjust the Si fingerprint geometrical parameters by using a different metal film thickness and composition.

With the right annealing duration, it is possible to achieve a ≈50% coverage on each of the four metal films, providing linewidths of ≈17 nm (9 nm Au film), 74 nm (11 nm Au film), 56 nm or 64 nm (9 nm Au Ag^−1^ film), and 137 nm (11 nm Au Ag^−1^ film). The 9 nm Au film provides the smallest linewidths, and as such, is the most promising sample for Industry. However, for now, it is challenging to control its dewetting in a standard laboratory setting. Based on these experimental results and requirements for the PUFs, the samples with a ≈50% coverage of nanostructured silicon were selected (the details of their fabrication are described in the Methods section) and tested for PUFs. Additionally, the Si‐fingerprint region can be selectively patterned using shadow‐masking (Figure S7, Supporting Information).

## 4 PUF Analysis

**Figure 4:**
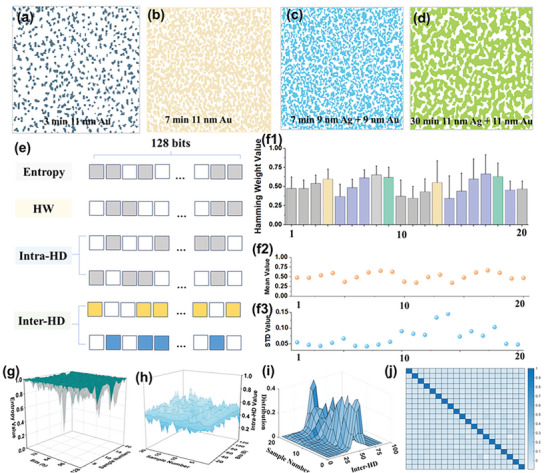
PUF analysis of the Si nanofingerprints. a–d) Transferred binary random matrix of four selected Si nanofingerprints, corresponding to the images shown in Figure 2c–f, with a matrix size of 128 × 128; e) Schematic illustration of the calculation methods; f1) Distribution of the Hamming weight (HW) for 20 selected samples; f2) Mean value and f3) standard deviation of the HW distribution for these 20 samples; g) Entropy value, h) intra‐Hamming distance (HD), i) inter‐HD, and j) the correlation coefficient of the 20 samples. More calculation details in Methods.

To quantitatively estimate the Si nanofingerprint randomness, the SEM images were converted into a 2D binary stochastic matrix by assigning digital “0” or “white” to the unoccupied pixels, i.e., flat Si, and digital “1” or “colour” to the occupied pixels, i.e., Si nanofingerprints, as shown in **Figure** **4**a–d, where different colours are used for different samples. The 2D binary image contains 128 × 128 pixels, corresponding to image sizes of either 1 × 0.8 µm^2^ or 4.2 × 5.7 µm^2^, which provide a CRP space of 2^16384^/µm^2^ and 2^16384^/25µm^2^ ≈  2^16380^/µm^2^, respectively. To check whether the spatial distribution of the large stochastic matrix is uniformly random or not, the 16384 bits are divided into 128 keys for calculations, each with a length of 128 bits. To assess the statistical strength, we compute the Hamming weight (HW) and entropy of each 2D key, the intra‐Hamming distance (intra‐HD) between 2D keys within the same PUF and the inter‐Hamming distance (inter‐HD), where the computation process is illustrated in Figure 4e, and described in more details in the Methods section. The HW evaluates the probability of “1” and “0” for each key. Twenty samples were investigated, identified with a sample number, and the synthetic conditions used for each sample are summarized in **Table** **2** (Methods section). The SEM images used for samples 1–9 were acquired at lower magnification (i.e., large SEM image size), and at higher magnification for the corresponding samples 10–18 (i.e., lower SEM image size). The HW value of each sample is shown in Figure 4‐f1. Si PUFs prepared using the same metal film are classified as one group, shown with a unique color in Figure 4a–d, f1. Most of the average HW values are close to the ideal value of 0.5 (Figure 4‐f2), similarly to the Si fingerprint coverage obtained via SEM analysis. For samples 10–18, the standard deviation (STD) is slightly higher than for samples 1–9 (Figure 4‐f3), indicating that higher magnification detection increases local variations on occupation rate. Shannon entropy is calculated for each sample and each key, to numerically evaluate the “uncertainty” of the inherent randomness levels.^[^ ^13^ ^]^ As shown in Figure 4g, most of the entropy is close to its ideal value, e.g. “1.” Some entropy loss corresponding to local areas without a high level of randomness do exist, seen in grey on Figure 4g. Furthermore, the intra‐HD and inter‐HD are used to estimate the similarity of each key within the same PUF, and between different PUFs, respectively. As shown in Figure 4h, the intra‐HD values are close to 0.5. Figure 4i shows the inter‐HD values of each PUFs. All the statistical curves follow Gaussian distributions, although samples10–18 exhibit wider distributions than samples 1–9. This is attributed to the larger deviation of HW that results from the use of higher magnification detection (more calculation details in Experimental Section, PUF calculation). Figure 4j shows the correlation coefficient colour map between the PUFs, which demonstrates a very low degree of similarity between the PUFs.

**Table 2:** Sample name and fabrication conditions used for PUF calculations.

Sample	Metal film	*t_d_* [min]	Image size	Sample	Metal film	*t_d_* [min]	Image size
1	9 nm Au	0.5	5.7 µm × 4.2 µm	10	9 nm Au	0.5	1.1 µm × 0.8 µm
2	9 nm Au	1	5.7 µm × 4.2 µm	11	9 nm Au	1	1.1 µm × 0.8 µm
3	9 nm Au	2	5.7 µm × 4.2 µm	12	9 nm Au	2	1.1 µm × 0.8 µm
4	11 nm Au	7	5.7 µm × 4.2 µm	13	11 nm Au	7	1.1 µm × 0.8 µm
5	9 nm Au + 9 nm Ag	5	5.7 µm × 4.2 µm	14	9 nm Au + 9 nm Ag	5	1.1 µm × 0.8 µm
6	9 nm Au + 9 nm Ag	7	5.7 µm × 4.2 µm	15	9 nm Au + 9 nm Ag	7	1.1 µm × 0.8 µm
7	9 nm Au + 9 nm Ag	10	5.7 µm × 4.2 µm	16	9 nm Au + 9 nm Ag	10	1.1 µm × 0.8 µm
8	9 nm Au + 9 nm Ag	15	5.7 µm × 4.2 µm	17	9 nm Au + 9 nm Ag	15	1.1 µm × 0.8 µm
9	11 nm Au + 11 nm Ag	30	5.7 µm × 4.2 µm	18	11 nm Au + 11 nm Ag	30	1.1 µm × 0.8 µm
19	9 nm Au + 9 nm Ag	5	5.7 µm × 4.2 µm	20	9 nm Au	5	5.7 µm × 4.2 µm

***t_d_***: dewetting duration.

## 5 BSE Detection

For practical applications, the nanofingerprint should be patterned within a small area next to the chip, which could be easily defined with a conventional lithography step. We demonstrate the compatibility of our approach to pattern micron‐sized regions with nanostructured silicon (≈8 nm linewidth) using a shadow mask during the metal deposition (Figure S7, Supporting Information): A unique Si fingerprint could be patterned at a specific location and fabricated by the OCM next to each microchip, before the procedures of the front‐end‐of‐line (FEOL), back‐end‐of‐line (BEOL) and packaging. Detection of Si fingerprints could be done routinely using secondary electron (SE) imaging within an SEM, a technique now widely accessible due to the availability of affordable bench‐top SEMs. However, SEs can only probe the top ≈10 nanometers of the sample surface and are thus highly sensitive to the surface quality and cleanliness. This complicates the use of SE imaging to investigate microchip surfaces that have been handled under real‐life conditions. Additionally, physical damage of unprotected Si fingerprints cannot be avoided. In fact, for practical applications, the Si fingerprint should be protected from its environment. This could be done using a polymer coating, such as those used for microchip packaging. To read/identify packaged Si fingerprints, PXCT would be the detection method of choice, thanks to its high resolution and ability to reconstruct 3D nanoscale objects. It is not, unfortunately, yet available to all research facilities.

**Figure 5:**
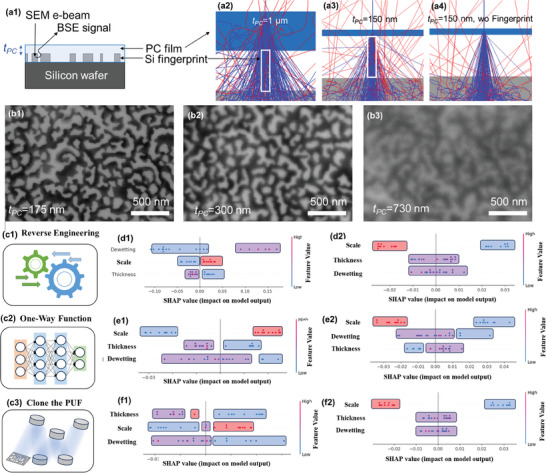
Detection of packaged Si nanofingerprints and potential risks of reverse engineering. a1) Schematic showing a cross‐section of the nanostructured Si surface protected with a polycarbonate film with a thickness *t_PC_* above the Si fingerprint. a2–a4) Monte Carlo simulation results showing electron trajectories in the sub‐surface region of a Si fingerprint covered with a polycarbonate film with a thickness *t_PC_* = 1 micron (a2) and *t_PC_* = 150 nm (a3). As a comparison, electron trajectories through a 150 nm‐thick polycarbonate film are shown in a region without a Si fingerprint (a4). b1–b3) Experimental BSE images of Si fingerprints protected with *t_PC_* = 175 nm (b1), *t_PC_* = 300 nm (b2) and *t_PC_* = 730 nm (b3), acquired at 20 kV (b1) and 30 kV (b2,b3). c1–c3) Scheme illustrating the potential risk of reverse engineering via model attack and following lithography process. d1,e1,f1) Mean values of Hamming weight, entropy and intra‐HD as output of the Shapley value and d2,e2,f2) standard deviations of Hamming weight, entropy and intra‐HD as output of the Shapley value, where the input features are dewetting time, metal thickness and SEM image size. The term “scale” represents the image SEM size input feature. (d–f) The red, blue and purple colored rectangles represent the positive/negative/mixed (contains both positive and negative) correlation between input and output features: Both the red and blue regions are predictable, while the purple region cannot be predicted.

Thus, we propose here a simpler alternative, based on the use of BSEs instead of SEs, which are able to probe nanostructures located up to ≈1 micron below the sample surface.^[^ ^27^ ^]^ To mimic the chip packaging, a polycarbonate film was spin‐coated above the nanostructured Si samples. Using accelerating voltages > 20 kV, we show that Si fingerprints protected with a polycarbonate coating thickness up to 300 nm can be imaged via BSE with high spatial resolution (**Figure** **5**a,b). We performed Monte Carlo simulations of the electron trajectories to investigate how the BSE emission yield η is expected to change as a function of the polymer thickness with and without a Si nanostruture (Figure 5a2–a4, more details in the Methods section). With a polycarbonate thickness *t_PC_* = 150 nm, η = 0.1853 ± 0.0042 on top of the Si fingerprint, and η = 0.1555 ± 0.0041 without the Si fingerprint. With *t_PC_* = 1 µm, η = 0.1266 ± 0.0042 and η = 0.1212 ± 0.0018 with and without the Si fingerprint respectively. Clearly, with a thin polymer film, e.g. *t_PC_* = 150 nm, the BSE emission yield is higher on top of the Si fingerprint, providing enough BSE contrast to image the nansotructrured Si. This is not the case when a thick polymer film is used, e.g. *t_PC_* = 1 µm, which leads to almost identical η over the different sample regions: BSE detection is not possible under these conditions. Additionally, the beam broadening induced by the added scattering through the polymer film is much more pronounced with *t_PC_* = 1 micron than with *t_PC_* = 150 nm (compare Figure 5a2 with Figure 5a3), which further contributes to the loss in spatial resolution observed with thicker protecting films. Overall, our Monte Carlo simulations align well with our experimental results that demonstrate high‐resolution BSE detection through a protecting polymer coating, provided *t_PC_* remains below a specific thickness.

## 6 Machine Learning Analysis

To verify whether the Si nanofingerprints are reliable or not for the AI modelling attacks, we adopt an aggressive attempt that places ourselves as attackers (Figure 5‐c1‐c3). The challenge‐response pairs can be verified by the OCM database to guarantee their authentication in the market. To reproduce the nanoscale Si‐PUF and fabricate a counterfeit via reverse engineering, the attacker needs to understand the formation mechanism and/or the physical models of the Si‐PUF. Whether the one‐way function from fabrication to PUF fabrication can be predicted via artificial intelligence, or the relationship between input physical synthesis features and output PUF features can be discovered is a critical question.^[^ ^28^ ^]^ Indeed, the attackers can only reproduce the nanoscale Si‐PUFs for illegal uses once they obtain such sensitive information. To our knowledge, no current available method or physical model are developed to accurately predict the correlation between input physical parameters and the PUF response outputs.

To further verify the security of the Si‐based nanofingerprint, we propose a machine learning analysis model to synergistically visualise the correlation between the input features, e.g., physical synthesis conditions, and Si fingerprint morphology, e.g. dimensions and patterns extracted from SEM (or BSE) images, and output features, e.g. entropy values and Hamming distances. This AI‐based method combines the random forest and Shapley values to visualize the importance and contribution of input physical features to the output PUF features.^[^ ^9^, ^29^ ^]^ The Shapley value is a powerful tool to visualise the correlation between input features and output features based on AI calculations. The red, blue and purple rectangles highlight regions corresponding to positive, negative and mixed (i.e., positive and negative) correlations, respectively (Figure 5d–f). Both the red and blue regions show predictability between the input and the output features, while the purple region correspond to unpredictable output features. 20 Si‐based nanoPUFs based on top‐view SEM images are fed into the AI tool, including dewetting time, metal film thickness, and SEM image size as input parameters, while the mean values and standard deviations of HW, entropy, and intra‐HD are defined as output parameters. Moreover, the rank shown in Figure 5d,f also exhibits the importance sequence of the input features to the output. As shown in Figure 5‐d1,d2,e1,e2,f1,f2, all the output parameters are affected the SEM image size: The image size is the most determining input parameter, while the dewetting time and metal film thickness have little influence, indicating that the formation of the nano Si‐PUF is highly unpredictable in regards to the metal deposition and dewetting step. The AI analysis results suggest that the Si‐based nanoPUF could be resilient to model attacks. Note that a higher amount of training samples would deliver more precise results. Thus, for practical applications, it is essential to carefully leverage both the SEM image size and the encoding capacity of the PUF. Moreover, even if the attacker discovers the exact Si fingerprint morphology, the reverse engineering would be too costly, requiring advanced deep‐UV lithography to clone or reproduce the 10 ‐100 nm linewidth of our Si‐based PUF. This could only be done by the OCM themselves.

## 7 Conclusion

Herein, we report a nanoscale Si‐based fingerprint for chip authentication, with linewidths tunable from ≈8 to 140 nm. The expected encoding capacity is high, e.g. 2^16384^ within ≈1 µm^2^ regions, which is both highly stochastic and space‐efficient, e.g. requires extremely low chip overhead. The Si nanofingerprint fabrication is CMOS‐compatible and requires four steps: metal deposition via sputtering, metal dewetting at 250 °C, metal‐assisted chemical etching in H_2_O_2_/HF and metal film dissolution. The random evolution of the metal dewetting, which is the entropy source of the Si fingerprint, is supported by molecular dynamic simulations. The inherent stochastic serpentine Si patterns were tested via HW, entropy, intra‐HD, inter‐HD, and similarity calculation: The one‐way function from the formation process to the random matrixes is resilient against machine learning attacks, making reverse engineering by counterfeiters costly and difficult, and thus, unlikely. For practical applications, detection of fingerprints protected with a polymer coating is demonstrated using standard BSE imaging, which is supported by Monte Carlo simulations.

## 8 Experimental Section

*Fabrication* ^[^ ^16^ ^]^: P‐type silicon wafers (<100>, resistivity 1–30 Ω cm, thickness 275 ± 25 *µm*, Si‐Mat, Germany) were cut into 1.5 × 1.5 cm^2^ pieces. The samples were pre‐treated via sonication in acetone for 5 min and oxygen plasma for 5 min (Quorum Emitech K1050X, 50 W, oxygen flow 30 mL min^−1^). Consequently, the Au and Au/Ag films were sputtered onto Si substrates using a Cressington Sputter Coater 108 auto at 40 mA for different durations. To minimize the deposition rate variation depending on the sample location in the chamber, the same location was targeted for each sample and each condition. The thickness of the metal films was estimated by SEM analysis and the coverage rate was calculated via top‐view SEM images and ImageJ. Dewetting process was carried out within a hot Nabertherm ashing furnace at 250 °C, ranging from 30 s to 30 min. The silicon nanofingerprints were synthesyized by immersing the metal‐coated Si substrates in a MACE solution containing 10 mL of H_2_O, 10 mL of HF, and 1 mL of H_2_O_2_ for various durations, and then rinsed three times in MilliQ water. The samples were then cleaned in a 20 mL of H_2_O and 4 mL of HF mixture for 5 min to remove any residual porous Si/ SiO_2_ that can be present at the nanostructured Si surface after MACE. After rinsing the samples three times in Milli‐Q water and once in ethanol, the samples were dried in air. The metal film was then dissolved in KI/I_2_ as previously described.^[^ ^16^ ^]^ It could also be dissolved in aqua regia if necessary.

### Molecular Dynamics Simulation

The simulation of metal dewetting is based on molecular dynamics LAMMPS platform.^[^ ^30^ ^]^ A 44 nm × 44 nm × 2 nm single crystal Si was initiated as substrate and a 1.6 nm SiO_2_ was formed onto it. Subsequently, 3 kinds of metal films were deposited, including 0.15 nm Au, 0.3 nm Au and 0.3 nm Ag over Au. The periodic boundary conditions were set within a simulation cell size of 44 nm × 44 nm × 21.6 nm. The interaction of Si─O, metal‐metal, and metal‐Si or metal‐O are described via Tersoff potential, embedded atom method, and Leonard Jones potential respectively, where σ_*Ag* − *O*_, ε_*Ag* − *O*_, σ_*Ag* − *Si*_, ε_*Ag* − *Si*_, σ_*Ag* − *Au*_, ε_*Ag* − *Au*_, σ_*Au* − *O*_, ε_*Au* − *O*_, σ_*Au* − *Si*_, ε_*Au* − *Si*_ are listed Table 1 below.

The Leonard Jones potential can be described as followed:^[^ ^25^ ^]^

(1) $$V\;\left(r\right)=4\varepsilon \left[{\left(\frac{\sigma }{r}\right)}^{12}-{\left(\frac{\sigma }{r}\right)}^{6}\right]$$

where *r* is the distance between two interacting atoms, σ is the distance at which the potential energy of the two interacting atoms is zero, and ε is the depth of the potential energy well.

The vacancy rate is set to 0.1.^[^ ^25^ ^]^ Conjugate gradient methods were utilized to minimize the system energy and reach the structural stable, where the stopping tolerance for energy is 10^−4^, and the stopping tolerance for force is 10^−10^ eV A^−1^. The temperature is initialized at 300K and increases to 523K at the first 10 ps. According to the recommended time length for each time evolution step based on the temperature‐damping parameters, the time evolution step is set as 1 fs. All the simulation results were demonstrated within OVITO software.

### Si Fingerprint Detection

SEM imaging was performed on a Zeiss Ultra Plus 55 field‐emission SEM. SE imaging was performed using the In‐Lens SE detector at an accelerating voltage of 5kV. BSE imaging was performed using an angular selective annular BSE detector located at below the objective lens and right above the sample holder, at an accelerating voltage of 20 and 30 kV. To investigate the influence of a protecting polymer film on SE and BSE imaging, a polycarbonate film was deposited via spin‐coating on top of the nanostructured Si sample. The thickness *t_PC_* of the polycarbonate film located above the Si fingerprint was estimated by performing cross‐sectional SEM analysis of a polycarbonate film spin‐coated on a flat Si surface (e.g., not etched via MACE). Films with *t_PC_* of 175, 300, and 730 nm were prepared by spin‐coating a dichloromethane solution with a concentration of polycarbonate of 25, 35, and 50 g L^−1^, respectively, at 6000 RPM for 30 s. The polycarbonate was generously provided by Sony DADC Inc.

### Monte Carlo Simulations

Monte Carlo simulations were performed using the freely available software Casino version 3.3.0.4 that calculates electron trajectories through objects of defined composition, density and dimensions. The Si wafer was simulated as a rectangular box of 40 × 40 × 9 µm^3^. The 9 µm thickness was chosen to be larger than the penetration depth of the 20 keV incident electrons. When required, a 200 nm wide and 1 thick µm Si fingerprint was located on top of the Si wafer. The polycarbonate film was simulated as a rectangular box of 40 × 40x*t_PC_* µm^3^, with a thickness *t_PC_*. The film scattering properties were set by the film composition, e.g. C_16_H_14_O_3_, whose density was set to 1.2. The electron beam diameter was set to 2 nm. 10 000 electron trajectories were simulated in each case, and only 200 electron trajectories were shown on the trajectory's maps. Electron energy was set to 20 keV. The BSE emission yield η was averaged over 10 Monte Carlo simulations for each configuration. The results of the 10 different simulations were always very close from each other, which is shown by the small standard deviation of the η values.

### Sample Selection for Calculation

The sample selection is shown in Table 2 below.

### PUF Calculations

The calculation includes Hamming weight, intra‐Hamming distance and inter‐Hamming distance.^[^ ^6^, ^31^ ^]^ To evaluate the quality of the Si nanofingerprint, the top‐view SEM images were binarized into a 128 × 128 matrix. For SEM image size of 5.7 µm × 4.2 µm^2^, each pixel (i.e., bit) has a spatial dimensions ranging from 32 to 46 nm, which is comparable to the linewidth of most the Si nanostructures synthesized in this work. For SEM image size of 1.1 µm × 0.8 µm^2^, each pixel has a dimension in the 6–8 nm range ≤ smallest Si nanostructure linewidth.

Hamming weight:

The Hamming weight of a key can describe the bit uniformity, which is given as:

(2) $$\mathrm{Uniformity}=\frac{1}{k}\sum_{i=1}^{k}R_{i}\left(j\right)\times 100\%$$

where *R_i_* (*j*) refers to the *j* ^th^ bit of a n‐bit key from the *i*th PUF and *k* is the total number of PUFs. In the current study, *k* is 20 and n is 128. The ideal value is 50%, which indicates that the numbers of bit ‘1′ and bit ‘0′ in a key is equal. Each key is selected via each row, and there are 128 rows.

Intra‐Hamming distance (intra‐HD)

The intra‐Hamming distance (intra‐HD) which is used to describe the stability and reproducibility denotes the difference among the keys generated from the same PUF in conditions. The intra‐HD is calculated by Equation:

(3) $$\mathrm{Intra}-\mathrm{HD}=\frac{1}{k}\sum_{i=1}^{k}\frac{1}{T}\sum_{l=1}^{T}\frac{HD\left(R_{i}^{1}\left(n\right),R_{i}^{l}\left(n\right)\right)}{k}\times 100\%$$

where $R_{i}^{l}\left(n\right)$ is the *n*‐bit response from the ith PUF at the *l*th row, *T* is the total number of the raw and *k* is the total number of PUFs. In the current study, *n* is 128 and *T* is also 128.

Between n‐bit responses of the ith cycle and the jth cycle from row i and j of a Si‐based PUF, where k is 128, q is 20.

Inter Hamming distance (inter‐HD)

The inter‐HD described the uniqueness among different PUFs, which could be calculated by:

(4) $$\mathrm{Inter}-\mathrm{HD}=\frac{2}{k\left(k-1\right)}\sum_{i=1}^{k-1}\sum_{j=i+1}^{k}\frac{1}{T}\sum_{l=1}^{T}\frac{HD\left(R_{i}^{l}\left(n\right),R_{j}^{l}\left(n\right)\right)}{k}\times 100\%$$

where $R_{i}^{l}\left(n\right)$ is the *n*‐bit response from the ith PUF at the *l*th row, *T* is the total number of the raw and *k* is the total number of PUFs. In the current study, In the current study, *k* is 20, *n* is 128 and *T* is 128.

The entropy value:

The Entropy could be derived from the probability of “1” and “0,” with the equation below:

(5) $$E=-\left[p\times log_{2}p+\left(1-p\right)log_{2}\left(1-p\right)\right]$$

where the *E* is the entropy and the p is the probability.

## Conflict of Interest

The authors declare no conflict of interest.

## Author Contributions

The original idea was conceived by B.L. and G.R.B., who also supervised the work. A.F. was responsible for depositing the metal films, conducting the dewetting experiments, performing shadow masking, and carrying out SEM imaging. A.F. and T.B. collaborated on the MACE experiments. B.L. oversaw the MD simulations, PUF calculation, and AI calculation, while G.B. conducted the Monte Carlo simulations. B.L., Y.Z., C.G., and S.F. collected techniques and information related to chip anti‐counterfeiting. B.L. prepared all the figures and the first draft of the manuscript, which was subsequently revised by B.L. and G.R.B., with contributions from A.F. and T.B.

## Supporting information



Supporting Information

## Data Availability

The data that support the findings of this study are available from the corresponding author upon reasonable request.
