# Supplementary material for: Nanoscale Silicon Fingerprints for Counterfeit Prevention in Microchips
Source: Small. 2025 Feb 4;21(10):2500878. doi: 10.1002/smll.202500878 (PMC11899515; doi:10.1002/smll.202500878)
Supplement: Supplementary file 1 — Supporting Information [file SMLL-21-2500878-s001.pdf]

NANO · MICRO
small

Supporting Information

for *Small*, DOI 10.1002/smll.202500878

Nanoscale Silicon Fingerprints for Counterfeit Prevention in Microchips

Bo Liu, Amin Farhadi, Theresa Bartschmid, Yamin Zhang, Chunsheng Guo, Shiwei Feng and Gilles R. Bourret**

Supporting Information

Nanoscale Silicon Fingerprints for Counterfeit Prevention in Microchips

Bo Liu, Amin Farhadi, Theresa Bartschmid, Yamin Zhang, Chunsheng Guo, Shiwei Feng and Gilles R. Bourret**

B. Liu, Y. Zhang, C. Guo, S. Feng
Faculty of Information Technology, College of Microelectronics, Beijing University of Technology, Beijing, 100124, People's Republic of China

A. Farhadi, T. Bartschmid, G. R. Bourret
Department of Chemistry and Physics of Materials, University of Salzburg, A-5020 Salzburg, Austria

Corresponding authors' e-mail address: boliu.ele@bjut.edu.cn and gilles.bourret@plus.ac.at

1. Note I Regional Contacts

Below are the contacts of the World Semiconductor Council (WSC) Anticounterfeiting Task Force (ACTF was established at 2012) for different regions:

SIA in China

Wenye Tan, Legal Counsel, Shanghai Silicon Intellectual Property Exchange Ltd.
wenyetan@gmail.com

SIA in Chinese Taipei

Dior Chen, Director, Semiconductor Industry Association in Chinese Taipei
dior@tsia.org.tw

SIA in Europe

Shane Harte, ESH Manager, Semiconductor Industry Association in Europe
shane.harte@eusemiconductors.eu

SIA in Japan

Teruhiko Sakaguchi, Deputy General Manager
Semiconductor Industry Association in Japan
teruhiko.sakaguchi@jeita.or.jp

SIA in Korea

Jong Wan Ko, General Manager, Semiconductor Industry Association in Korea
jwko@ksia.or.kr

SIA in US
Devi Keller, Director of Global Policy, Semiconductor Industry Association
dkeller@semiconductors.org
More information on the WSC is available at http://www.semiconductorcouncil.org.

2. Note II The Counterfeit Chips: Definition

Regarding the counterfeit chips, it is necessary to provide a brief introduction of the counterfeited chips, detection method and avoidance techniques as shown in Figure S1.

First of all, what is the Counterfeit Chip? 1) unauthorised copy, such as via reverse engineering; 2) not reach the standards of OCM design, model, or performances; 3) not produced via OCM or authorised channel; 4) defective or aged OCM product sold or considered as "new"; 5) false marking or remarking with forged information (one of the most prevalence counterfeited methods).

3. Note III The Counterfeit Chips: Taxonomy

Detailly, the counterfeited chip could be classified as follows:

Recycled and Remark: Recycled chips are usually taken from the used printed circuit boards, then repackaged and sold to the market as new.

Overproduction: unauthorized access to the designer's IP and fabricated chip without a contract.

Defective: under unauthorized access or overproduction, the chip may be out-of-spec or defective.

Cloning: this can be achieved in two ways: reverse engineering or access IP illegally.

Forged: false certification of standards, programs or logs.

Tampered: this is most under die level or package level, e.g., the hardware Trojan.

4. Note IV The Counterfeit Chips: Detection

Generally speaking, the counterfeit detection method could be briefly classified into three categories, physical, electrical and aging. A basic purpose of those detection methods is to judge whether the chip is counterfeited, without destroying the original chip. Moreover, essential details about the chip, e.g. the company logo, manufacturing data, and identification should be recorded. The electrical method is to check the electrical function of the chip; the ageing inspection is to estimate the lifetime and degradation of the chip; the physical inspection is to obtain the material, fabrication or packaging information.

The physical inspection could be further classified into four categories: 1) incoming inspection, e.g. the low-power visual inspection (LPVI) to obtain the basic information of packaging, shipping, lot and data code. This method could detect counterfeit remarking or recycling; 2) external tests, such as scanning electron microscopy (SEM) or scanning acoustic microscopy (SAM) to find

defective, anomalous or out-of-spec sites; 3), the interior test should decap the chip to check the internal structure, wire pull, die shear and so on; 4) material analysis include the penetrating detection tools such as the X-ray, Fourier transform IR spectroscopy (FTIR) and energy dispersive spectroscopy (EDS) and so on.

The terahertz time-domain spectroscopy could check the authentication of packaged integrated circuits to reveal whether unexpected materials are implemented[1]. The advanced X-ray could also check the packaged integrated circuits. For example, the damaged secure digital memory cards could be detected via X-ray equipment[2].

5. Note V The Counterfeit Chips: Avoidance Techniques

CDIR sensors: The combating die and IC recycling (CDIR) sensor was developed to prevent recycling, e.g., utilizing an anti-fuse memory block so that the recycling process could not alter the usage time.

Secure Split Test (SST): The SST is based on random numbers and modern asymmetry cryptographic algorithms such as RSA, to maintain the security of design houses and their IPs.

Hardware Metering: This is a protocol that allows the design house to control the IC after fabrication, such as adding a locked state of the chip until identified by the IP holder with the primary input sequence.

Split Manufacturing: Separation the fabrication of front end of line (FEOL) and back end of line (BEOL) into different foundries to disperse and alleviate the risk of IP theft or overproduction.

IC Camouflaging: Adding dummy contacts to puzzle or against the reverse engineering.

Hardware Watermarking: The hardware watermarking is to create a unique pattern that can not be replicated to protect the IPs.

Physical Unclonable Function (PUF) : PUF utlizes the intrinsic variations of the chip to endow unique and unclonable responses of each hardware.

Package ID: A unique tagging that do not require access the designs.

6. Note VI The Brif of Electronic based PUF for Chip

In 2002, when the physical one-way function was invented, a CMOS-compatible arbiter PUF (APUF) was proposed[3]. The APUF yields a large challenge-response pair (CRP) space by harnessing the random interconnect and transistor gate time delays. The SRAM PUF was then invented, which does not require an extra dedicated layout[4]. These two categories of silicon-based PUF, including their upgraded designs, i.e. ROPUF, latch PUF and flip-flop PUF, can be implemented on field-programmable gate array (FPGA) platforms[5]. However, they inevitably lose some entropy due to the nearly flawless foundry process involved in their manufacturing. Such an entropy loss may result in the inherent vulnerability of modelling attacks and brute force attacks from a higher computing power. Additionally, the susceptibility of the PUF response to thermal noise,

environmental variation and ageing, increases the probability that the responses become more predictable. The emerging CMOS-compatible nanotechnologies, such as memristors, carbon-nanotube field-effect transistors (CNFETs), and two-dimensional materials-based electronic devices could provide plenty of reconfigurable entropy sources[6–8]. Although promising, high production costs and some unsolved technical issues have prevented their use in Industry[9]: For example, the safe and remote update of the reconfigurable CRP used in reconfigurable PUFs (RPUFs) by a trusted third party is still complex, costly, resource-intensive, and space-intensive, and as such is a major source of concern.

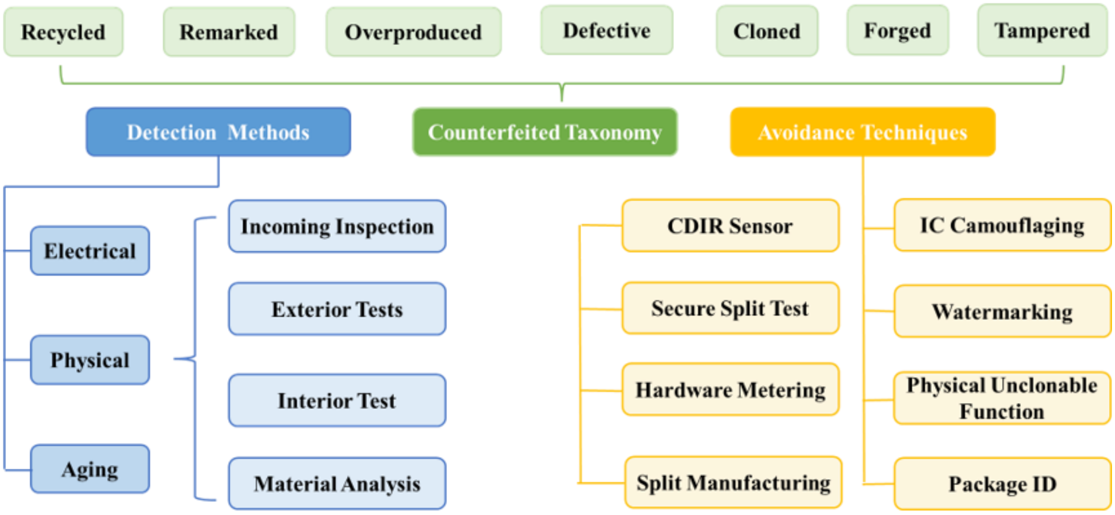

Figure S1 (a) brief classification of the counterfeited taxonomy, detection method and avoidance techniques for counterfeited chip. Note that all the data collected from the ERAI 2023 annual report: https://www.erai.com/erai_blog/3183/2023_annual_report

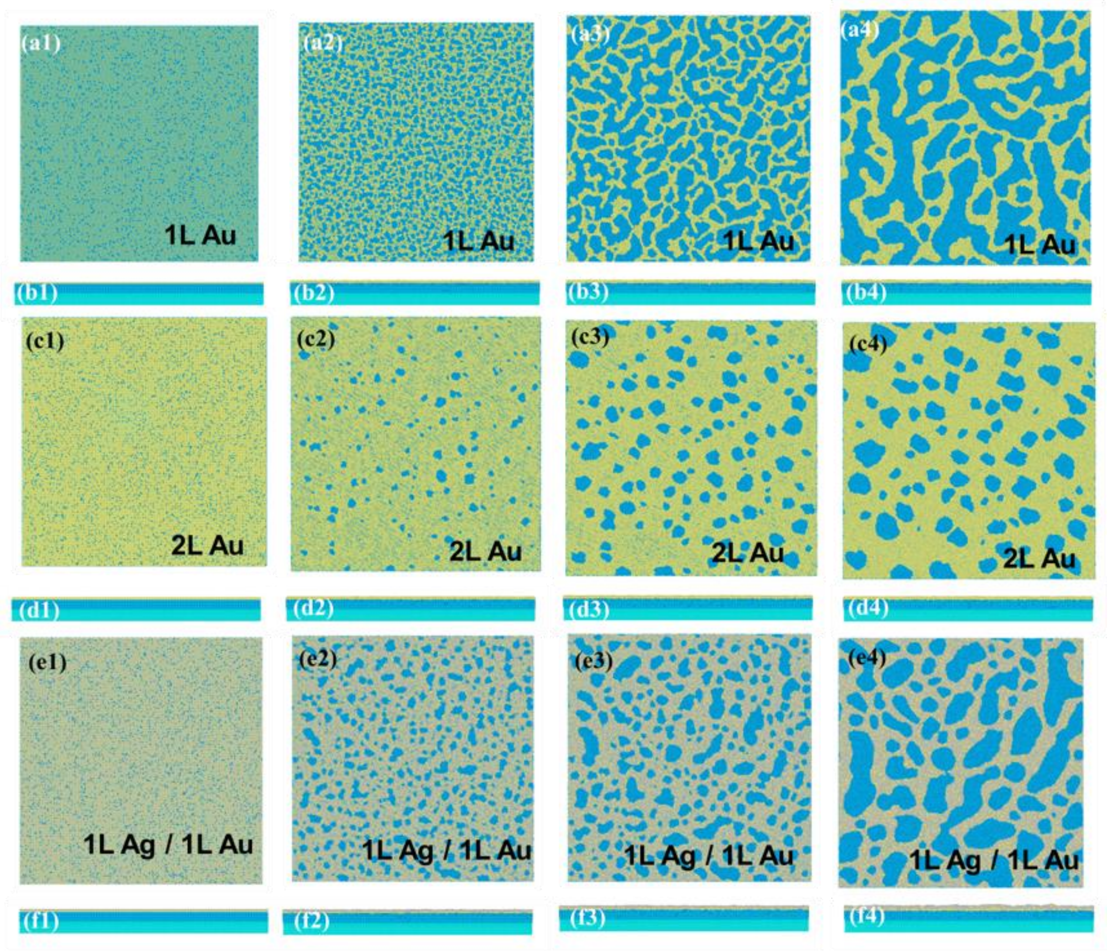

Figure S2 The time evolution of the metal dewetting at Si/SiO_2 substrate. (a), (c) and (e) are the top-views. (b), (d), and (e) are the cross-sectional views.

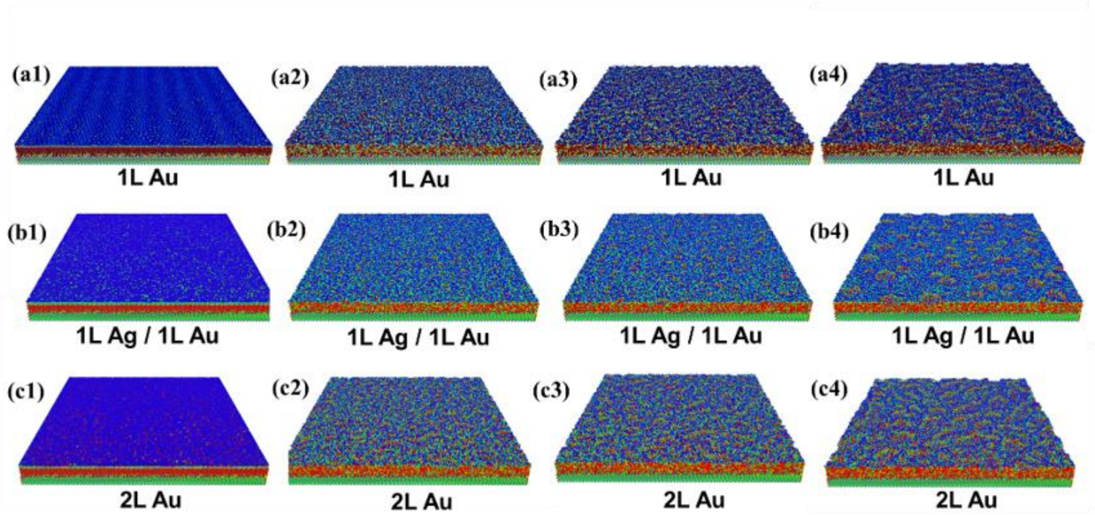

Figure S3 The tension revolution of the metal dewetting at Si/SiO_2 substrate in 3D view.

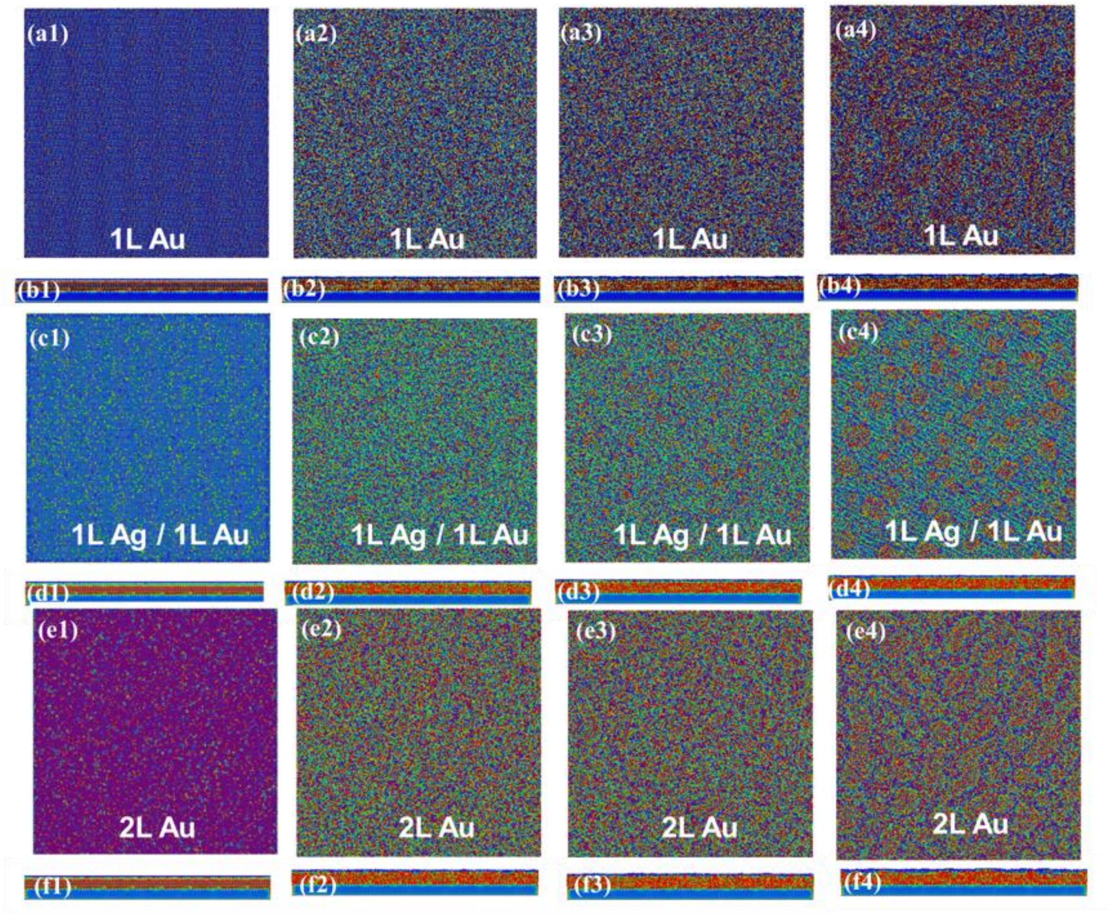

Figure S4 The tension revolution of the metal dewetting at Si/SiO_2 substrate in front and cross-sectional view.

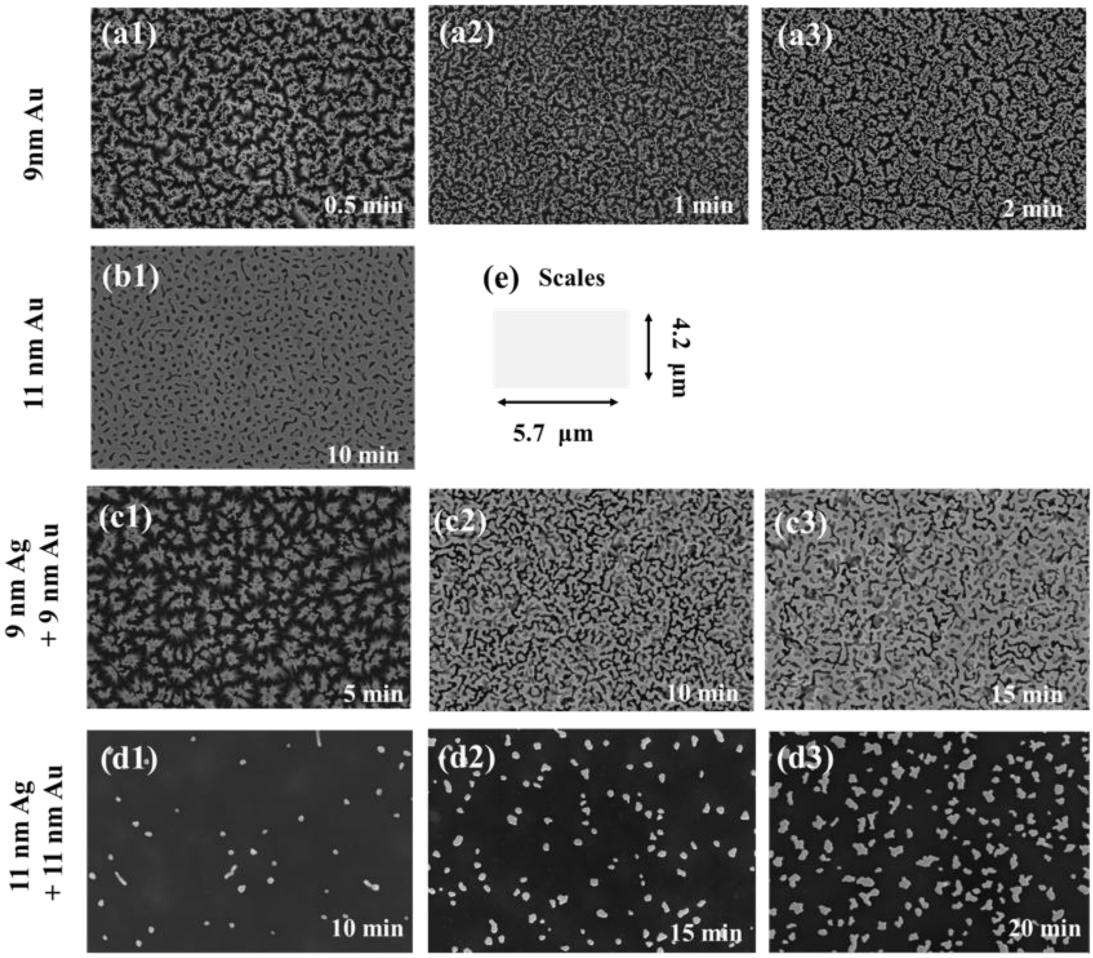

Figure S5 SEM images of four kinds of samples (a-d) with different dewetting times, where (e) is the total image size.

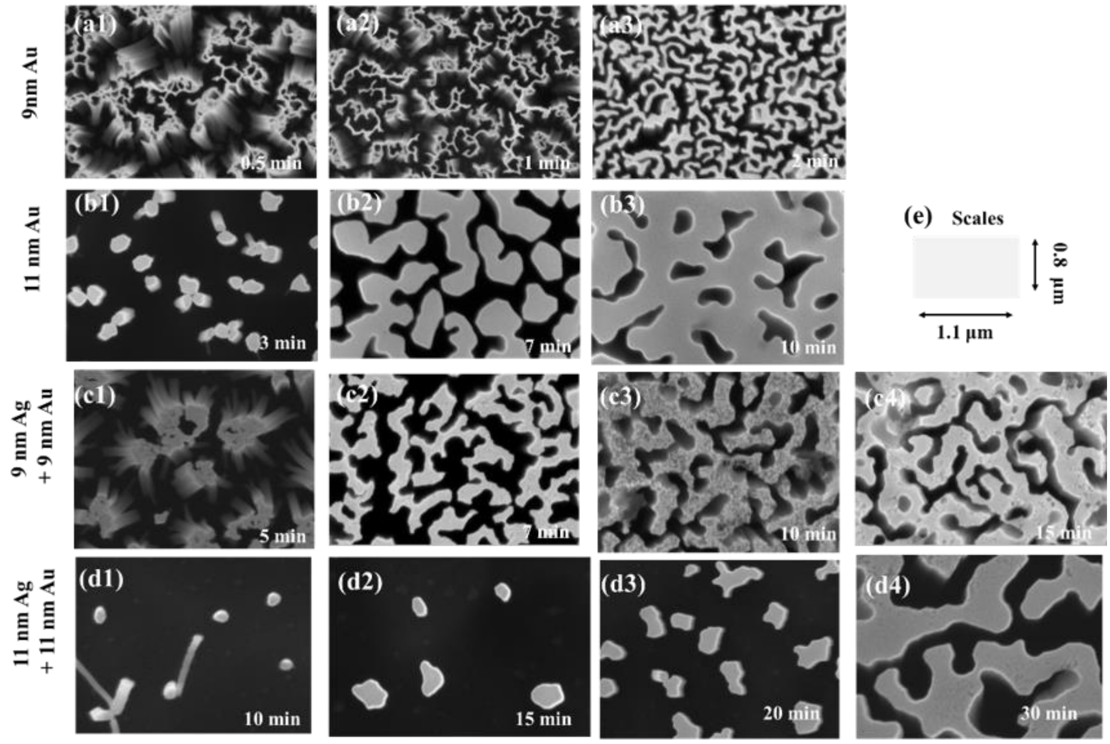

Figure S6 SEM images of four kinds of samples (a-d) with different dewetting times, where (e) is the total image size.

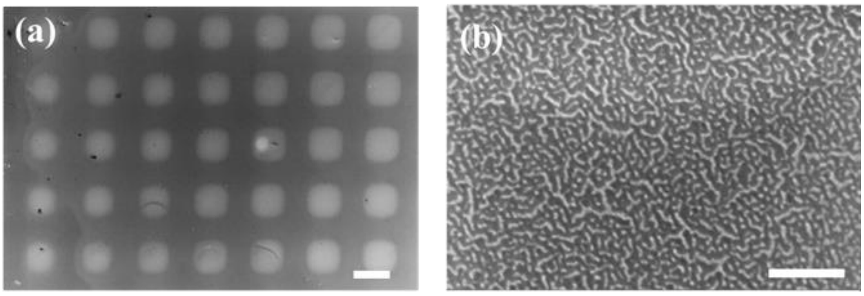

Figure S7 (a-b) Top-view SEM image of the sample under shadow masking. (a) the Au micron-sized patterns prepared *via* shadow-masking, imaged before dewetting and MACE, and used to pattern the Si nanofingerpinrt regions, scale bar 50 µm (b) Top-view SEM image of the Si nanofingerpinrt oproduced after MACE and metal film dissolution in one of the pattern shown in (a), scale bar 200 nm.

7. References.

[1] K. Ahi, S. Shahbazmohamadi, N. Asadizanjani, *Opt. Lasers Eng.* **2018**, *104*, 274.
[2] F. Thomas-Brans, T. Heckmann, K. Markantonakis, D. Sauveron, *IEEE Access* **2022**, *10,* 33742.
[3] B. Gassend, D. Clarke, M. Van Dijk, S. Devadas, *Proc. ACM Conf. Comput. Commun. Secur.* **2002**, 148.
[4] D. E. Holcomb, W. P. Burleson, K. Fu, *IEEE Trans. Comput.* **2009**, *58*, 1198.
[5] S. D. Suh, G Edward, *Proc. 44th ACM Annu. Des. Autom. Conf. (ACM).* **2007**, 9.
[6] B. Gao, B. Lin, Y. Pang, F. Xu, Y. Lu, Y. C. Chiu, Z. Liu, J. Tang, M. F. Chang, H. Qian, H. Wu, *Sci. Adv.* **2022**, *8*, eabn7753.
[7] A. Dodda, S. Subbulakshmi Radhakrishnan, T. F. Schranghamer, D. Buzzell, P. Sengupta, S. Das, *Nat. Electron.* **2021**, *4*, 364.
[8] L.-M. P. & Z. Z. Donglai Zhong, Jingxia Liu, Mengmeng Xiao, Yunong Xie, Huiwen Shi, Lijun Liu, Chenyi Zhao, Li Ding, *Nat. Electron.* **2022**, *5*, 424.
[9] Y. Gao, D. C. Ranasinghe, S. F. Al-Sarawi, O. Kavehei, D. Abbott, *IEEE Access* **2016**, *4*, 61.
